# Transferrin Impacts* Bacillus thuringiensis* Biofilm Levels

**DOI:** 10.1155/2016/3628268

**Published:** 2016-11-29

**Authors:** Bianca Garner, Elrica Brown, Martha Taplin, Angel Garcia, Baracka Williams-Mapp

**Affiliations:** ^1^Tougaloo College, 500 West County Line Road, Tougaloo, MS, USA; ^2^Jackson State University, 1400 John R. Lynch Street, Jackson, MS, USA

## Abstract

The present study examined the impact of transferrin on* Bacillus thuringiensis* biofilms. Three commercial strains, an environmental strain (33679), the type strain (10792), and an isolate from a diseased insect (700872), were cultured in iron restricted minimal medium. All strains produced biofilm when grown in vinyl plates at 30°C.* B. thuringiensis* 33679 had a biofilm biomass more than twice the concentration exhibited by the other strains. The addition of transferrin resulted in slightly increased growth yields for 2 of the 3 strains tested, including 33679. In contrast, the addition of 50 *μ*g/mL of transferrin resulted in an 80% decrease in biofilm levels for strain 33679. When the growth temperature was increased to 37°C, the addition of 50 *μ*g/mL of transferrin increased culture turbidity for only strain 33679. Biofilm levels were again decreased in strain 33679 at 37°C. Growth of* B. thuringiensis *cultures in polystyrene resulted in a decrease in overall growth yields at 30°C, with biofilm levels significantly decreased for 33679 in the presence of transferrin. These findings demonstrate that transferrin impacts biofilm formation in select strains of* B. thuringiensis*. Identification of these differences in biofilm regulation may be beneficial in elucidating potential virulence mechanisms among the differing strains.

## 1. Introduction

*Bacillus thuringiensis *is a ubiquitous Gram-positive, spore forming microbe. Because it is employed as a biological pesticide,* B. thuringiensis* is routinely isolated from agricultural commodities [1–5]. This microbe produces insecticidal toxins that target select hosts [6, 7]. Taxonomically,* B. thuringiensis *falls within the* Bacillus cereus sensu lato* group of microbes, which include the human opportunistic pathogen* B. cereus *and the zoonotic pathogen* Bacillus anthracis* [8]. Although a high degree of genetic similarity is found among the three strains,* B. thuringiensis* is considered a distinct species based on the physiological variations, including virulence factors [9].

Biofilm development is one of the many physiological processes that differ within the* B. cereus sensu lato* group microbes. The ability to form biofilms is not aligned with a particular species in the group and can fluctuate from strain to strain [10]. Biofilm formation is also not correlated with cell surface moieties or pathogenicity [10, 11]. In* B. cereus *clinical strains, microbes recovered from certain niches, like the oral cavity, are less likely to produce biofilms in cultures. This phenomenon has not been recorded in* B. thuringiensis *[10, 12]. What is evident is that the ability of* B. cereus* to form biofilms is contingent on environmental conditions, including nutrient accessibility [13].

Required in low levels for normal cellular functions, iron has been verified to regulate biofilm formation in countless microbes, including* Haemophilus influenzae, Klebsiella pneumonia*, and* Escherichia coli *[14–16]. Iron is required for biofilm development in* Staphylococcus aureus, Pseudomonas aeruginosa*, and* Mycobacterium smegmatis *[17–19]. The iron impact cannot be attributed to growth unaided, as the increase is more dramatic in biofilms than in planktonic cells [20]. In* B. cereus*, the impact of iron on biofilm formation is highly variable. Only two (2)* B. cereus* strains demonstrated an increase in biofilm when iron was restricted, versus nine (9) strains that had a decrease in biofilm when iron was limited [21]. In the nine (9) strains that required iron for biofilm growth, biofilm biomass directly correlated to the specific iron source utilized for growth. For example, only two (2) strains exhibited biofilm concentrations higher in the presence of transferrin than in the iron restricted samples [21]. These findings are in direct opposition to reports that transferrin increases* B. cereus sensu lato* microbial growth under iron restriction [22–24].

The present study sought to investigate the impact of transferrin on* B. thuringiensis *biofilm physiology. Commercial strains originally isolated from different sources were assayed for biofilm formation in minimal medium. Turbidity was monitored in microtiter plates to ascertain whether the addition of transferrin impacted culture growth. Biofilm levels were quantified to classify the impact of transferrin on biofilm biomass of the three different strains.

## 2. Materials and Methods

### 2.1. Microbiological Conditions

*B. thuringiensis *strains were purchased from the American Type Culture Collection (ATCC). Strains were randomly selected but included the type strain (laboratory) and an environmental strain. Strains were maintained as spores on sporulation agar (23 g nutrient agar, 0.5 g yeast extract, 6.0 mg MnCl_2_, 95.0 mg MgCl_2_, and 78.0 mg CaCl_2_ per liter) at 4°C. Growth assays were carried out in a defined minimal medium consisting of MM9 salts (3 g KH_2_PO_4_, 5 g NaCl, and 10 g NH_4_Cl per liter), glucose (0.2%), and casamino acids (0.3%). This medium was treated with Chelex-100 (Bio-Rad) to remove excess iron and filter sterilized. Metals were added to this sterile medium base at the following concentrations: 830 *μ*M Mg, 36 *μ*M Mn, and 0.32 *μ*M Zn. This defined medium is referred to hereafter as CTM for Chelex-treated medium. Holo-transferrin (Sigma) was purchased in powder form and solubilized in water. Transferrin was filter sterilized and added to the cultures in the indicated amounts.

### 2.2. Biofilm Measurement

Quantification of biofilm biomass was performed with the microtiter plate assay as previously described with some modifications [21]. Brain-heart infusion agar (Fisher Scientific) slants were inoculated for overnight incubation at 37°C. Overnight cultures were then used to inoculate CTM at final turbidity (OD_600_) of 0.02 in 96-well microtiter plates (200 *μ*L). Samples were incubated overnight, without shaking. After incubation, growth was measured via the turbidity (*A*_620_) with the BioTek Synergy 2 plate reader. After measuring the turbidity, the plates were inverted to remove unattached cells. Adherent cells were stained with crystal violet (200 *μ*L) and samples were incubated for at least 1 minute. Plates were then inverted to remove excess dye and washed thrice with phosphate buffered saline. Plates were inverted a final time and remained inverted for 5 minutes. Gram stain alcohol decolorizer (200 *μ*L) was added to each well to solubilize the crystal violet bound to the adherent cells. The optical densities of these samples are the biofilm biomass and were measured spectrophotometrically at* A*_540_ with the BioTek Synergy 2. Biofilm level was calculated as the biomass of the sample divided by the biomass of the sample without transferrin. Biofilm level indicates either an increase in biofilm (>1.0) or a decrease in biofilm (<1.0) as compared to the transferrin-free samples.

### 2.3. Statistical Analysis

Experiments were repeated 3 times. Data is the average of the measurements and error bars, where indicated, are the standard deviation. A standard *t*-test was performed on data and significance established with *p* < 0.05.

## 3. Results and Discussions

Production of biofilms has been well documented in Gram-positive microbes, including the* Bacillus* species. Elucidation of the mechanisms that govern biofilm formation in* B. cereus *group microbes has implications both in the environment, where they are frequent contaminants, and in medicine, where nosocomial infections are linked to various species [12]. Iron availability has been linked to biofilm formation in numerous microbes and has emerged as an area of interest in* B. cereus sensu lato *group because of its implications in microbial virulence [24].

Biofilm formation is altered by transferrin in some* B. cereus *laboratory and environmental strains [21]. Although* B. anthracis, B. cereus*, and* B. thuringiensis* share many genetic similarities, the regulatory patterns that govern physiology vary among the microbes. Thus, while some* B. cereus *strains may require iron for optimal biofilm conditions, it is unclear how iron or any of the potential iron sources will impact biofilm formation in* B. thuringiensis*. This member of the* B. cereus sensu lato* group has traditionally not been viewed as pathogenic, as many of the toxin genes target insects, as opposed to humans. There are cases, however, of human infections by* B. thuringiensis *in the immunocompromised, with some infections characterized by biofilm development [25–27]. The basis for the present study was to determine whether transferrin would impact* B. thuringiensis *biofilm levels similar to the process observed in* B. cereus*.

Commercially available* B*.* thuringiensis* strains were purchased from the American Type Culture Collection (ATCC) to examine the potential role of transferrin in biofilm development under iron limiting conditions [28]. ATCC strain 33679, serotype H3:3a,3b, is a commonly used strain that encodes the insecticidal toxins and was originally isolated from a diseased insect larva. ATCC strain 700872, serovar israelensis, is an environmental strain isolated from soil. ATCC strain 10792 is the type strain originally isolated from animal tissue. Thus, the three strains selected represent differing origins of isolation. While this has not been demonstrated to be a determining factor in the ability of* B. thuringiensis* to form biofilms, it does provide a comparison of how isolates might respond to transferrin when cultured in defined, minimal medium.

CTM containing MM9 was selected as the medium for biofilm assays.* B. cereus *demonstrates an iron restrictive phenotype when cultured in MM9 alone [22]. MM9 supplemented with additional nutrients are more effective for the growth of* B. anthracis *[29]. CTM in the present study contains MM9, glucose, and casamino acids. This minimal medium was then Chelex-treated to remove all metal contamination, which allows for control of the metal composition in the medium. Although there might be some internal carryover iron from the overnight growth,* B*.* thuringiensis *cells grown in CTM for 24 hours are iron restricted based on molecular and biochemical analyses of cultures [30].

**Figure 1:**
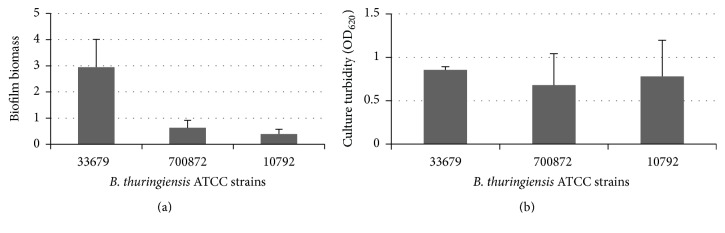
(a)* B. thuringiensis* strains form biofilms in iron restrictive medium. CTM was inoculated to an OD_600_ of 0.02 in vinyl plates and incubated for 24 hours at 30°C. Microtiter plates were inverted to remove unattached cells. Samples were stained with crystal violet and washed with PBS. Biofilm biomass was detected by the addition of alcohol decolorizer, which was measured at* A*_540_. Data are presented as the average, with error bars indicating the standard deviation. (b)* B. thuringiensis* strains grow in iron restrictive medium. CTM was inoculated to an OD_600_ of 0.02 in vinyl plates and incubated for 24 hours at 30°C. Culture turbidity was measured at OD_620_. Samples are presented as the average, with error bars indicating the standard deviation.

Biofilm biomass was measured in strains cultured in vinyl plates at 30°C for 24 hours in CTM. All three strains produced biofilm under the conditions tested. Biofilm formation was the highest in strain 33679 and the lowest in strain 10792 (Figure 1(a)). Biofilm biomass in strain 33679 was more than three (3) times higher than in strain 10792 and two (2) times higher than in strain 700872. These results are consistent with studies which indicate that biofilm concentration varies within a given species and demonstrate* B. thuringiensis *biofilm formation in minimal medium [31].

Microbial growth was measured spectrophotometrically in the vinyl plates to identify whether differences in biofilm could be correlated with differences in culture growth. All strains grew to similar levels in CTM, with optical densities above 0.5 (Figure 1(b)). Strain 33679 demonstrated the highest growth yields, but there was no significant difference in final growth yields between the three strains. Thus, differences observed in biofilm biomass between the three strains cannot be attributed solely to culture growth levels.

While iron is an abundant metal required in only small quantities for normal cellular processes, microbes cannot readily access iron during an infection. Transferrin is one of several host iron binding proteins utilized for growth by a variety of microbes [32]. In* B. cereus, *the addition of transferrin to iron restrictive medium results in an increase in growth [22]. Growth yields and biofilm levels were measured for the three strains cultured in the presence of transferrin for 24 hours in vinyl plates at 30°C to observe whether the compound altered* B. thuringiensis *physiology.

**Figure 2:**
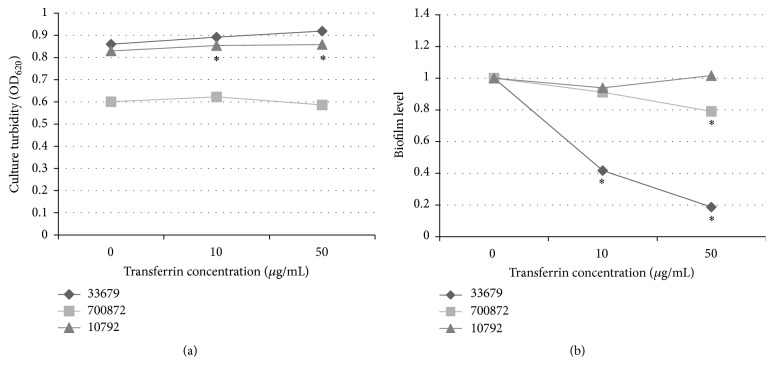
(a)* B. thuringiensis* strains grew in the presence of transferrin. CTM with varying concentrations of transferrin was inoculated to an OD_600_ of 0.02 in vinyl plates and incubated for 24 hours at 30°C. Culture turbidity was measured in a plate reader at OD_620_. Samples are presented as the average. Asterisks indicate a statistically significant (*p* < 0.05) change as compared to the no transferrin samples. (b) Transferrin decreases biofilm formation in* B. thuringiensis* strains. CTM with varying concentrations of transferrin was inoculated to an OD_600_ of 0.02 in vinyl plates and incubated for 24 hours at 30°C. Samples were analyzed for biofilm biomass as described. Biofilm levels indicate a change in biomass relative to the baseline. Asterisks indicate a statistically significant (*p* < 0.05) change as compared to the no transferrin samples.

The addition of transferrin resulted in increased growth yields for 2 of the 3* B. thuringiensis* strains when compared to the no transferrin cultures. A statistically significant dose-dependent increase in growth was observed for strain 10792 with transferrin (Figure 2(a)). An increase in growth was observed for 33679, but the values were not statistically significant when compared to the no transferrin samples. Only strain 700872 demonstrated a decrease in growth with 50 *μ*g/mL of transferrin, but only by approximately 3%. Thus, as in* B. cereus*, transferrin can increase growth of some, but not all,* B. thuringiensis *strains.

The vast differences in biofilm biomass concentrations made comparison between the three strains difficult, as the levels for 2 of the strains were considerably lower in the minimal medium (Figure 1(a)). Thus, the change in biofilm concentration was measured as a ratio of the biofilm level. This allowed for a comparison of the impacts of transferrin on biofilm development between the strains. For this analysis, the samples without transferrin were designated the baseline. Biofilm biomass of the transferrin containing cultures was measured spectrophotometrically. Each biomass was then divided by the baseline. Thus, biofilm levels indicate either a decrease (below 1.0) or an increase (above 1.0) in concentration relative to the baseline.

While the addition of transferrin resulted in slight increases in growth for 2 of the 3 strains, transferrin had the opposite impact on biofilm levels. Transferrin resulted in a decrease in biofilm formation for 2 strains cultured in vinyl plates at 30°C for 24 hours. A statistically significant dose-dependent decrease in biofilm level was observed in strain 33679 (Figure 2(b)). Biofilm levels decreased by more than 50% when strain 33679 was cultured with 10 *μ*g/mL of transferrin and 80% with 50 *μ*g/mL of transferrin as compared to the baseline (Figure 2(b)). Strain 700872 had an 8% decrease in biofilm level with 10 *μ*g/mL of transferrin and a significant 20% decrease when cells were cultured with 50 *μ*g/mL of transferrin. Although a slight decrease in biofilm level occurred for strain 10792 with 10 *μ*g/mL of transferrin, no overall transferrin trend was measured. These studies suggest that while* B. thuringiensis *culture growth is increased with transferrin for some strains, the biofilm levels may not be regulated in a similar manner. Because environmental factors can alter biofilm biomass, it was postulated that the impact of transferrin on biofilm levels is dependent on additional factors.

Like many microbes,* Bacillus *species alter their physiology to adapt to the changing environment, allowing them to survive diverse conditions. Temperature is a critical environmental regulator of* B. cereus sensu lato* physiology [33]. While 37°C is the preferable temperature for* B. cereus* and* B. anthracis* virulence, lower temperatures are important in the pathogenicity for the insect pathogen* B. thuringiensis *[33, 34]. Although the optimal growth temperature for* B. thuringiensis *is indicated to be 30°C, the organism has been isolated from human infections and, thus, can grow at higher temperatures.

**Figure 3:**
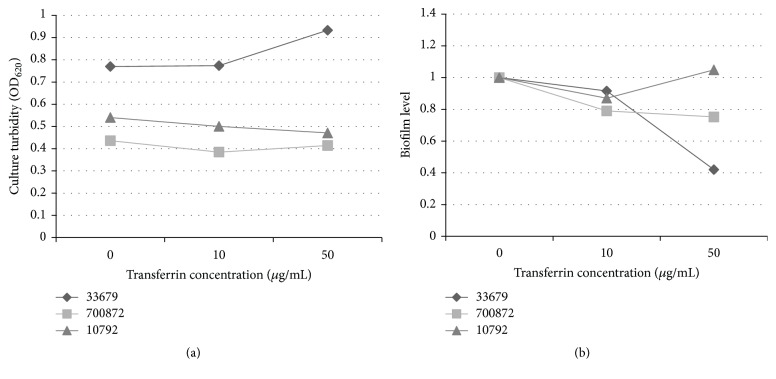
(a)* B. thuringiensis* strains grow in vinyl plates at 37°C. CTM was inoculated to an OD_600_ of 0.02 and incubated for 24 hours at 37°C. Culture turbidity was measured in a plate reader at OD_620_. Samples are presented as the average. (b) Transferrin decreases biofilm formation in certain* B. thuringiensis* strains in vinyl plates at 37°C. CTM was inoculated to an OD_600_ of 0.02 and incubated for 24 hours at 37°C. Samples were analyzed for biofilm biomass as described. Biofilm biomass was detected by the addition of alcohol decolorizer. Biofilm levels indicate a change in biomass relative to the baseline. Samples are presented as the average.

To ascertain whether temperature is important in the transferrin impact on biofilm formation,* B. thuringiensis *strains were cultured in vinyl plates at 37°C for 24 hours and assayed as described above. Growth was measured first to determine whether the change in temperature altered growth. Strain 33679 had the highest yields, similar to the values observed at 30°C (Figure 3(a)). In contrast, growth for strains 700872 and 10792 at 37°C was less than growth observed at 30°C. The addition of 50 *μ*g/mL of transferrin resulted in increased growth for 33679 and decreased growth for both 700872 and 10792 as compared to the no transferrin samples, but the results were not statistically significant.

Biofilm was evident in all* B. thuringiensis *strains cultured at 37°C in vinyl plates. The addition of 10 *μ*g/mL of transferrin resulted in a slight decrease in biofilm levels for all strains (Figure 3(b)). The increase of transferrin to 50 *μ*g/mL resulted in additional decreases for strains 33679 and 700872. The decrease in biofilm levels for strain 33679 was more than 80% and more than 20% for 700872. These responses were similar to biofilm levels observed at 30°C, indicating that transferrin impacts biofilm development in vinyl plates, regardless of the temperature for some* B. thuringiensis *strains.

Temperature is one environmental factor that may play a role in biofilm formation. In* B. cereus, *strains demonstrated a higher affinity to form biofilms based on the growth substrate. The iron regulation of biofilm formation was more evident when these microbes were cultured in polystyrene plates versus other substrates [21]. To examine whether polystyrene was an effective surface for biofilm formation,* B. thuringiensis* strains were cultured as described above in noncoated polystyrene microtiter plates for 24 hours at 30°C.

**Figure 4:**
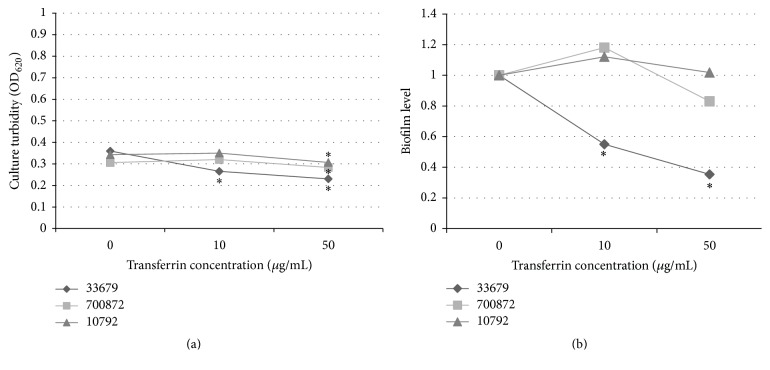
(a)* B. thuringiensis* strains form biofilms in polystyrene plates. CTM was inoculated to an OD_600_ of 0.02 and incubated in CTM for 24 hours at 30°C. Culture turbidity was measured in a plate reader at OD_620_. Samples are presented as the average. Asterisks indicate a statistically significant (*p* < 0.05) change as compared to the no transferrin samples. (b) Transferrin decreases biofilm formation in certain* B. thuringiensis* strains in polystyrene plates. CTM was inoculated to an OD_600_ of 0.02 and incubated for 24 hours at 30°C. Samples were analyzed for biofilm biomass as described. Biofilm levels indicate a change in biomass relative to the baseline. Asterisks indicate a statistically significant (*p* < 0.05) change as compared to the no transferrin samples.

Growth was decreased for all strains in polystyrene plates when compared to growth in vinyl plates without the added transferrin. No culture turbidity average was over 0.5, with all three strains exhibiting growth between optical densities of 0.3 to 0.4 (Figure 4(a)). It is unclear what role polystyrene had in growth, as levels were more than 50% decreased when compared to growth in vinyl plates at the same temperature. A dose-dependent statistically significant decrease in growth was observed for strain 33679 in the presence of transferrin (Figure 4(a)). The addition of 50 *μ*g/mL of transferrin resulted in a statistically significant decrease in growth for all three strains.

Regardless of the low growth levels, biofilm was detected in all three strains cultured at 30°C in polystyrene. The addition of transferrin to* B. thuringiensis* 33679 resulted in a statistically significant decrease in biofilm levels similar to those observed in the vinyl cultures (Figure 1 and Figure 4(b)). Transferrin increased biofilm levels in strains 700872 and 10792 at 10 *μ*g/mL, but the addition of 50 *μ*g/mL resulted in a decrease of biofilm levels back to or below the baseline. Thus, the transferrin impact on* B. thuringiensis* 33679 is consistent at 30°C, regardless of the growth substrate.

Biofilms are environmentally important niches that allow* B. cereus *microbes to survive under diverse and harsh conditions. These microbes must use a variety of cues to regulate the production of the various factors that help to establish and sustain biofilms. Once established, biofilms represent an important physiological state where increased microbial survival occurs. These are the first studies to demonstrate a potential role for transferrin in* B. thuringiensis* biofilm development. Within the* B. cereus sensu lato *group,* B. thuringiensis* has traditionally not been viewed as pathogenic, as many of the identified* B. thuringiensis* toxin genes target insects. There are cases, however, of human infections by* B. thuringiensis *generally from nosocomial infections [32–34]. Thus, it is important to identify factors that facilitate biofilm formation in this strain that is a potential health hazard.

The present study highlights the complex regulatory mechanism that governs biofilm levels in* B. thuringiensis*. Biofilm formation is determined by the physiological state of the microbe, which is dependent on many environmental variables. The ability of transferrin to impact biofilm formation was highly variable and dependent on multiple factors. Additional comparative biochemical and molecular studies must be performed to identify the regulatory pathways that govern biofilm formation within the species. The discovery of these processes may potentially lead to the identification of additional virulence factors within the* B. cereus* group.

## 4. Conclusions

Transferrin can impact biofilm levels in some* B. thuringiensis* strains cultured under minimal conditions. This process may be altered, however, by growth temperature and growth substrate.
